# Supplementary material for: Disease and Participant-Related Correlates of Genetic Testing Completion for Hereditary Eye Disorders in a Cohort of over 1400 Patients
Source: Ophthalmol Sci. 2026 May 8;6(7):101218. doi: 10.1016/j.xops.2026.101218 (PMC13292590; doi:10.1016/j.xops.2026.101218)
Supplement: Supplemental Figure 1 [file mmc1.pdf]

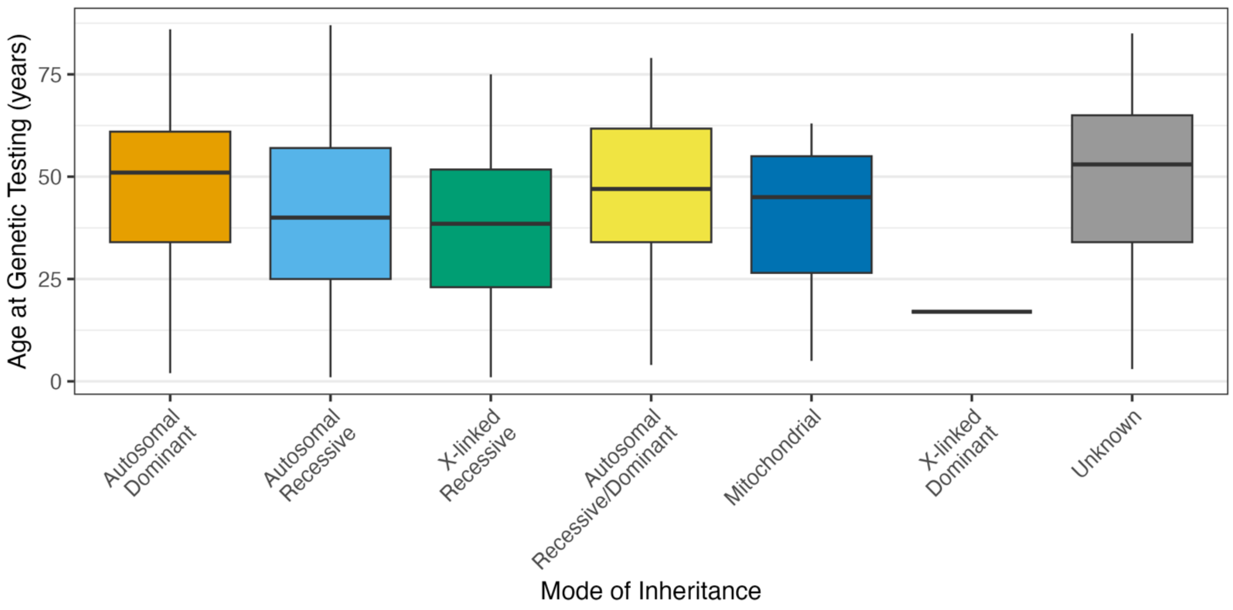

Supplemental Figure 1. Age at genetic testing completion by mode of inheritance. Boxplots display median, interquartile range, and range across major inheritance categories (n=1087). 'Unknown' denotes cases where inheritance pattern remained undetermined after testing.
