# Supplementary material for: Disease and Participant-Related Correlates of Genetic Testing Completion for Hereditary Eye Disorders in a Cohort of over 1400 Patients
Source: Ophthalmol Sci. 2026 May 8;6(7):101218. doi: 10.1016/j.xops.2026.101218 (PMC13292590; doi:10.1016/j.xops.2026.101218)
Supplement: Supplemental Table 2 [file mmc3.pdf]

Supplemental Table 2. Putative clinical diagnoses and genetic testing outcomes in the full genetic eye disease cohort (n=1466).

Putative clinical diagnosis	Age of symptom onset	Age at presentation	Freq	(+)-GT	I-GT	(-)-GT	Untested	Genes identified in participants with likely molecular diagnosis[a]
Retinitis Pigmentosa	25 (14-42)	46 (32-56)	473	44% (207)	18% (86)	13% (61)	25% (119)	*USH2A* (32), *RHO* (25), *RP1* (16), *RPGR* (12), *EYS* (10), *PRPF31* (9), *PRPH2* (8), *MAK* (7), *CRB1* (6), *PDE6B* (6), *NR2E3* (5), *CNGA1* (4), *SNRNP200* (4), *TULP1* (4), *BBS1* (3), *CLN3* (3), *FAM161A* (3), *FLVCR1* (3), *IMPDH1* (3), *PROM1* (3), *RPE65* (3), *CEP290* (2), *IFT140* (2), *NRL* (2), *PRPF8* (2), *ABCA4* (1), *BBS2* (1), *BEST1* (1), *C8ORF37* (1), *CERKL* (1), *CLRN1* (1), *CNGB1* (1), *EXOSC5* (1), *GNPTG* (1), *HGSNAT* (1), *IMPG1* (1), *IMPG2* (1), *MT-TP* (1), *NPHP4* (1), *PDE6A* (1), *PDE6G* (1), *PRPF3* (1), *PRPS1* (1), *RBP3* (1), *RLBP1* (1), *RP2* (1), *SAG* (1), *SCAPER* (1), *SLC52A2* (1), *SPATA7* (1), *TOPORS* (1), *VPS13B/COH1* (1)
Stargardt disease	25 (12-40)	38 (21-53)	255	71% (182)	12% (31)	3% (7)	14% (35)	*ABCA4* (172), *PRPH2* (6), *ABCA4+PRPH2* (2), *FZD4* (1), *PROM1* (1)
Pattern Dystrophy	60 (47-69)	60 (50-68)	106	15% (16)	11% (12)	18% (19)	56% (59)	*PRPH2* (12), *IMPG2* (2), *ARMS2* (1), *MT-TS2* (1)
Cone Dystrophy	38 (15-56)	48 (32-62)	101	18% (18)	25% (25)	22% (22)	36% (36)	*CRB1* (2), *GUCA1A* (2), *GUCY2D* (2), *RDH12* (2), *CACNA2D4* (1), *CNGB3* (1), *CTNNA1* (1), *FAM161A* (1), *KCNV2* (1), *MFSD8/CLN7* (1), *PROM1* (1), *RP1* (1), *RP2* (1), *RPGR* (1)
Cone-Rod dystrophy	30 (18-44)	44 (31-58)	99	39% (39)	29% (29)	12% (12)	19% (19)	*CRX* (10), *PROM1* (3), *RPGR* (3), *SCA7* (3), *ABCA4* (2), *CACNA1F* (2), *CERKL* (2), *PRPH2* (2), *CEP78* (1), *CLN1* (1), *CNGA3* (1), *CRB1* (1), *GUCY2D* (1), *KIZ* (1), *PRCD* (1), *RET* (1), *RHO* (1), *RPE65* (1), *TTLL5* (1), *USH2A* (1)
Usher syndrome	18 (12-30)	34 (20-50)	67	84% (56)	0% (0)	6% (4)	10% (7)	*USH2A* (31), *PCDH15* (7), *ADGRV1* (5), *CDH23* (5), *MYO7A* (4), *USH1C* (2), *CLRN1* (1), *GPR98* (1)
Hereditary optic neuropathy	19 (9-39)	33 (14-42)	42	31% (13)	19% (8)	26% (11)	24% (10)	*OPA1* (4), *ACO2* (3), *MT-ND4* (3), *AFG3L2* (1), *MT-ND1* (1), *MT-ND6* (1)
Unspecified macular dystrophy	38 (25-44)	42 (31-56)	39	10% (4)	18% (7)	28% (11)	44% (17)	*CDH3* (1), *CRB1* (1), *PRPH2* (1), *TULP1* (1)
Best disease (e.g. Bestrophinopathy & Best vitelliform macular dystrophy)	20 (12-35)	37 (28-54)	33	64% (21)	3% (1)	3% (1)	30% (10)	*BEST1* (20), *PRDM13* (1)
Oculocutaneous Albinism	0 (0-3)	22 (6-37)	25	32% (8)	24% (6)	4% (1)	40% (10)	*OCA2* (4), *GPR143* (2), *TYR* (2)
L-ORD (late-onset retinal degeneration)	62.5 (56-70)	68 (61-77)	24	12% (3)	17% (4)	21% (5)	50% (12)	*C1QTNF5* (3)
Achromatopsia	0.5 (0-9)	16 (5-37)	22	59% (13)	9% (2)	18% (4)	14% (3)	*CNGA3* (7), *CNGB3* (5), *KCNV2* (1)
Retinoschisis	10 (5-39)	34 (18-46)	21	48% (10)	10% (2)	5% (1)	38% (8)	*RS1* (9), *OPA1* (1)
Unspecified retinal dystrophy	44.5 (26-55)	47 (27-64)	20	10% (2)	40% (8)	15% (3)	35% (7)	*MT-ATP6* (1), *NPHP1* (1)
Choroideremia	20.5 (13-41)	39 (33-57)	18	89% (16)	6% (1)	6% (1)	0% (0)	*CHM* (16)
Leber Congenital Amaurosis/Severe early-onset retinal dystrophy (LCA/SEORD)	0 (0-2)	9 (3-29)	17	59% (10)	6% (1)	18% (3)	18% (3)	*CEP290* (2), *CRB1* (2), *CABP4* (1), *LCA5* (1), *NPHP4* (1), *RPE65* (1), *RPGRIP1* (1), *SPATA7* (1)

Putative clinical diagnosis	Age of symptom onset	Age at presentation	Freq	(+)-GT	I-GT	(-)-GT	Untested	Genes identified in participants with likely molecular diagnosis[a]
Stickler syndrome	11 (2-36)	20 (5-40)	12	50% (6)	25% (3)	17% (2)	8% (1)	*COL2A1* (4), *Col11A1* (1), *LRP2* (1)
Mitochondrial retinal dystrophy	38 (36-42)	45 (41-49)	9	78% (7)	11% (1)	11% (1)	0% (0)	*MT-TL1* (6), *RHO* (1)
Occult macular dystrophy	25 (13-53)	48 (32-63)	9	44% (4)	0% (0)	33% (3)	22% (2)	*RP1L1* (4)
Pseudoxanthoma elasticum	53 (38-61)	64 (44-72)	9	22% (2)	33% (3)	0% (0)	44% (4)	*ABCC6* (2)
Bardet-Biedl Syndrome (Laurence-Moon)	13 (8-24)	27 (12-34)	7	100% (7)	0% (0)	0% (0)	0% (0)	*BBS1* (5), *BBS2* (1), *BBS4* (1)
Congenital Stationary Night Blindness	10 (6-28)	12 (6-34)	7	57% (4)	29% (2)	0% (0)	14% (1)	*NYX* (3), *CACNA1F* (1)
Adult-onset vitelliform macular dystrophy	60 (60-67)	62 (62-69)	5	0% (0)	20% (1)	20% (1)	60% (3)	
Blue Cone Monochromacy	0 (0-3)	13 (8-15)	5	100% (5)	0% (0)	0% (0)	0% (0)	*OPN1LW/OPN1MW* (3), *OPN1LW* (1), *OPN1MW* (1)
Gyrate Atrophy	5.5 (3-11)	32 (27-43)	4	50% (2)	0% (0)	0% (0)	50% (2)	*OAT* (2)
Hereditary retinal vascular disorder	32.5 (10-56)	36 (18-57)	4	0% (0)	50% (2)	0% (0)	50% (2)	
Nyctalopia	46 (30-59)	44 (27-61)	4	0% (0)	25% (1)	25% (1)	50% (2)	
SCA7-related retinal dystrophy (Spinocerebellar ataxia 7)	19.5 (13-31)	28 (21-36)	4	75% (3)	0% (0)	0% (0)	25% (1)	*SCA7* (3)
Joubert syndrome	1 (0-2)	2 (2-3)	3	33% (1)	0% (0)	33% (1)	33% (1)	*C5orf42* (1)
LCHAD-related retinitis pigmentosa	5 (2-10)	4 (2-17)	3	100% (3)	0% (0)	0% (0)	0% (0)	*HADHA* (3)
North Carolina macular dystrophy	9 (7-39)	7 (6-44)	3	67% (2)	0% (0)	0% (0)	33% (1)	*PRDM13* (2)
Alport syndrome-related retinal dystrophy	39 (30-48)	34 (28-40)	2	50% (1)	0% (0)	0% (0)	50% (1)	*COL4A5* (1)
Alstrom syndrome	0 (0-0)	19 (19-19)	2	100% (2)	0% (0)	0% (0)	0% (0)	*ALMS1* (2)
Biette Crystalline Corneoretinal Dystrophy	36.5 (35-38)	48 (41-54)	2	100% (2)	0% (0)	0% (0)	0% (0)	*CYP4V2* (2)
Central areolar choroidal dystrophy (CACD)	27 (15-39)	30 (17-44)	2	50% (1)	0% (0)	0% (0)	50% (1)	*PRPH2* (1)
Enhanced S-Cone Syndrome	12 (8-16)	18 (17-20)	2	100% (2)	0% (0)	0% (0)	0% (0)	*NR2E3* (2)
Batten disease	5 (5-5)	5 (5-5)	1	100% (1)	0% (0)	0% (0)	0% (0)	*CLN3* (1)
Incontinentia pigmenti	0 (0-0)	17 (17-17)	1	100% (1)	0% (0)	0% (0)	0% (0)	*IKBKG* (1)
Methylmalonic acidemia-related retinal dystrophy	0 (0-0)	21 (21-21)	1	0% (0)	0% (0)	0% (0)	100% (1)	
Sorsby fundus dystrophy	53 (53-53)	48 (48-48)	1	0% (0)	100% (1)	0% (0)	0% (0)	
Von Hippel-Lindau syndrome	18 (18-18)	30 (30-30)	1	100% (1)	0% (0)	0% (0)	0% (0)	*VHL* (1)
Zellweger spectrum disorder	0 (0-0)	1 (1-1)	1	100% (1)	0% (0)	0% (0)	0% (0)	*PEX10* (1)

Putative clinical diagnosis	Age of symptom onset	Age at presentation	Freq	(+)-GT	I-GT	(-)-GT	Untested	Genes identified in participants with likely molecular diagnosis[a]

This table summarizes the putative clinical diagnoses assigned at presentation, their frequencies, and corresponding genetic testing (GT) outcomes. For each phenotypic category, the percentage of participants with positive (likely molecular diagnosis), inconclusive, negative, or no genetic testing is reported. Identified causative genes are listed for participants with likely molecular diagnoses in each category.

[a]Note: The number of genes by diagnosis may not match the total number of participants with likely molecular diagnoses, as full genetic testing reports were unavailable for some cases. However, likely molecular diagnosis was confirmed in the medical record for these individuals.
