# Supplementary material for: Disease and Participant-Related Correlates of Genetic Testing Completion for Hereditary Eye Disorders in a Cohort of over 1400 Patients
Source: Ophthalmol Sci. 2026 May 8;6(7):101218. doi: 10.1016/j.xops.2026.101218 (PMC13292590; doi:10.1016/j.xops.2026.101218)
Supplement: Supplemental Table 3 [file mmc4.pdf]

Supplemental Table 3. Comparison of demographic and clinical characteristics by genetic testing status. Data presented as % (n) or median (IQR).

Parameter	Completed genetic testing (N=1088)	Untested (N=378)	Raw p-value	Adjusted p-value
**Sex**			χ^2 p=0.903	1
Male	48% (524)	48% (180)		
Female	52% (564)	52% (198)		
**Age**	50 (IQR 34-66) years	60 (IQR 44-73) years	M-W p=6.7e-11	1.1e-09*
**Race**				
White	65% (704)	52% (198)	χ^2 p=3.3e-05	4.3e-04*
Black or African American	20% (219)	35% (131)	χ^2 p=1.7e-08	2.4e-07*
Asian	8% (84)	4% (17)	χ^2 p=0.08	0.8
Native Hawaiian or Other Pacific Islander	<1% (1)	<1% (1)	Fisher's p=0.449	1
American Indian or Alaska Native	<1% (3)	1% (2)	Fisher's p=0.608	1
Other	7% (77)	8% (29)	χ^2 p=1	1
**Ethnicity**			χ^2 p=0.662	1
Hispanic or Latino	5% (51)	4% (15)		
Not Hispanic or Latino	95% (1037)	96% (363)		
**Age of symptom onset**	25 (IQR 10-43) years	37 (IQR 16-55) years	M-W p=1.1e-10	1.7e-09*
**Age at presentation**	41 (IQR 25-55.2) years	49 (IQR 35-63) years	M-W p=5.4e-11	9.2e-10*
**Symptom duration prior to presentation**	6 (IQR 1-19) years	4 (IQR 1-20) years	M-W p=0.029	0.31
**Duration of follow-up**	6 (IQR 2-12) years	5 (IQR 1-12) years	M-W p=0.003	0.035*
**Baseline BCVA, better-seeing eye**	0.301 (IQR 0.097-0.699)	0.204 (IQR 0.097-0.602)	M-W p=0.234	1
**Baseline BCVA, worse-seeing eye**	0.477 (IQR 0.176-1)	0.477 (IQR 0.176-1)	M-W p=0.535	1
**Follow-up BCVA, better-seeing eye**	0.477 (IQR 0.097-1)	0.398 (IQR 0.097-0.903)	M-W p=0.257	1
**Follow-up BCVA, worse-seeing eye**	0.699 (IQR 0.301-1.301)	0.699 (IQR 0.204-1.301)	M-W p=0.869	1

*Adjusted p<0.05 (Holm-Bonferroni correction for multiple comparisons)
Abbreviations: *BCVA* = best-corrected visual acuity (logMAR); *IQR* = interquartile range; *M-W* = Mann-Whitney U test; χ^2 = chi-square test; *Fisher's* = Fisher's exact test.
