# Supplementary material for: Disease and Participant-Related Correlates of Genetic Testing Completion for Hereditary Eye Disorders in a Cohort of over 1400 Patients
Source: Ophthalmol Sci. 2026 May 8;6(7):101218. doi: 10.1016/j.xops.2026.101218 (PMC13292590; doi:10.1016/j.xops.2026.101218)
Supplement: Supplemental Table 5 [file mmc6.pdf]

Supplemental Table 5. Multivariable logistic regression results for factors associated with molecular diagnostic yield (likely molecular diagnosis vs. inconclusive or negative results). LASSO regression selected sex, race, age of symptom onset, and follow-up BCVA in the better-seeing eye as predictors.

Variable	Level	OR (95% CI)	P-value
Sex (ref: Female)	Male	1.37 (1.05-1.78)	**0.02**
Race (ref: White)	American Indian/Alaska Native	0.77 (0.07-16.98)	0.84
	Asian	0.68 (0.41-1.13)	0.13
	Black or African American	0.37 (0.26-0.51)	**<0.001**
	Native Hawaiian/Pacific Islander	56308.57 (0-NA)	0.97
	Other race	0.58 (0.35-0.98)	**0.04**
Age of onset, years (ref: 0-9)	10-19	1.03 (0.67-1.59)	0.88
	20-29	0.8 (0.51-1.28)	0.35
	30-39	0.7 (0.44-1.13)	0.14
	40-49	0.52 (0.33-0.82)	**0.005**
	50-59	0.2 (0.12-0.33)	**<0.001**
	60-69	0.2 (0.11-0.35)	**<0.001**
	70+	0.12 (0.04-0.32)	**<0.001**
Follow-up BCVA, better eye	Follow-up BCVA, better eye (logMAR)	1.33 (1.08-1.66)	**0.008**

Predictor selection performed using LASSO regression with 10-fold cross-validation (lambda.1se). Ethnicity, baseline BCVA (better- and worse-seeing eye), and follow-up BCVA in the worse-seeing eye were evaluated but not retained by LASSO. Reference categories: Female (sex), White (race), 0-9 years (age of onset). Abbreviations: *BCVA* = best-corrected visual acuity (logMAR); *OR* = odds ratio; *CI* = confidence interval.
