# Supplementary material for: Disease and Participant-Related Correlates of Genetic Testing Completion for Hereditary Eye Disorders in a Cohort of over 1400 Patients
Source: Ophthalmol Sci. 2026 May 8;6(7):101218. doi: 10.1016/j.xops.2026.101218 (PMC13292590; doi:10.1016/j.xops.2026.101218)
Supplement: Supplemental Table 6 [file mmc7.pdf]

Supplemental Table 6. Causative genes identified among participants with likely molecular diagnoses.

Gene	Inheritance Pattern	Frequency	% of participants
ABCA4	Autosomal Recessive	177	26.2%
USH2A	Autosomal Recessive	64	9.5%
PRPH2	Autosomal Dominant	32	4.7%
RHO	Autosomal Dominant	27	4%
BEST1	Autosomal Recessive/Dominant	21	3.1%
RP1	Autosomal Recessive/Dominant	17	2.5%
CHM	X-linked Recessive	16	2.4%
RPGR	X-linked Recessive	16	2.4%
CRB1	Autosomal Recessive	12	1.8%
CRX	Autosomal Dominant	10	1.5%
EYS	Autosomal Recessive	10	1.5%
PRPF31	Autosomal Dominant	9	1.3%
RS1	X-linked Recessive	9	1.3%
BBS1	Autosomal Recessive	8	1.2%
CNGA3	Autosomal Recessive	8	1.2%
PROM1	Autosomal Recessive/Dominant	8	1.2%
MAK	Autosomal Recessive	7	1%
NR2E3	Autosomal Recessive	7	1%
PCDH15	Autosomal Recessive	7	1%
CNGB3	Autosomal Recessive	6	0.9%
MT-TL1	Mitochondrial	6	0.9%
PDE6B	Autosomal Recessive	6	0.9%
SCA7	Autosomal Dominant	6	0.9%
ADGRV1	Autosomal Recessive	5	0.7%
CDH23	Autosomal Recessive	5	0.7%
OPA1	Autosomal Dominant	5	0.7%
RPE65	Autosomal Recessive	5	0.7%
TULP1	Autosomal Recessive	5	0.7%

Gene	Inheritance Pattern	Frequency	% of participants
CEP290	Autosomal Recessive	4	0.6%
CLN3	Autosomal Recessive	4	0.6%
CNGA1	Autosomal Recessive	4	0.6%
COL2A1	Autosomal Dominant	4	0.6%
FAM161A	Autosomal Recessive	4	0.6%
MYO7A	Autosomal Recessive	4	0.6%
OCA2	Autosomal Recessive	4	0.6%
RP1L1	Autosomal Dominant	4	0.6%
SNRNP200	Autosomal Dominant	4	0.6%
ACO2	Unknown after genetic testing	3	0.4%
C1QTNF5	Autosomal Dominant	3	0.4%
CACNA1F	X-linked Recessive	3	0.4%
CERKL	Autosomal Recessive	3	0.4%
FLVCR1	Autosomal Recessive	3	0.4%
GUCY2D	Autosomal Dominant	3	0.4%
HADHA	Autosomal Recessive	3	0.4%
IMPDH1	Autosomal Dominant	3	0.4%
IMPG2	Autosomal Recessive	3	0.4%
MT-ND4	Mitochondrial	3	0.4%
NYX	X-linked Recessive	3	0.4%
OPN1LW/OPN1 MW	X-linked Recessive	3	0.4%
PRDM13	Autosomal Dominant	3	0.4%
ABCC6	Autosomal Recessive	2	0.3%
ALMS1	Autosomal Recessive	2	0.3%
BBS2	Autosomal Recessive	2	0.3%
CLRN1	Autosomal Recessive	2	0.3%
CYP4V2	Autosomal Recessive	2	0.3%
GPR143	X-linked Recessive	2	0.3%
GUCA1A	Autosomal Dominant	2	0.3%

Gene	Inheritance Pattern	Frequency	% of participants
IFT140	Autosomal Recessive	2	0.3%
KCNV2	Autosomal Recessive	2	0.3%
NPHP4	Autosomal Recessive	2	0.3%
NRL	Autosomal Recessive	2	0.3%
OAT	Autosomal Recessive	2	0.3%
PRPF8	Autosomal Dominant	2	0.3%
RDH12	Autosomal Recessive	2	0.3%
RP2	X-linked Recessive	2	0.3%
SPATA7	Autosomal Recessive	2	0.3%
TYR	Autosomal Recessive	2	0.3%
USH1C	Autosomal Recessive	2	0.3%
AFG3L2	Autosomal Dominant	1	0.1%
ARMS2	Autosomal Recessive	1	0.1%
BBS4	Autosomal Recessive	1	0.1%
C5orf42	Autosomal Recessive	1	0.1%
C8ORF37	Autosomal Recessive	1	0.1%
CABP4	Autosomal Recessive	1	0.1%
CACNA2D4	Autosomal Recessive	1	0.1%
CDH3	Autosomal Recessive	1	0.1%
CEP78	Autosomal Recessive	1	0.1%
CLN1	Autosomal Recessive	1	0.1%
CNGB1	Autosomal Recessive	1	0.1%
COL4A5	X-linked Recessive	1	0.1%
CTNNA1	Autosomal Dominant	1	0.1%
Col11A1	Autosomal Dominant	1	0.1%
EXOSC5	Autosomal Recessive	1	0.1%
FZD4	Unknown after genetic testing	1	0.1%
GNPTG	Autosomal Recessive	1	0.1%
GPR98	Autosomal Recessive	1	0.1%
HGSNAT	Autosomal Recessive	1	0.1%

Gene	Inheritance Pattern	Frequency	% of participants
IKBKG	X-linked Dominant	1	0.1%
IMPG1	Autosomal Recessive	1	0.1%
KIZ	Autosomal Recessive	1	0.1%
LCA5	Autosomal Recessive	1	0.1%
LRP2	Autosomal Recessive	1	0.1%
MFSD8/CLN7	Autosomal Recessive	1	0.1%
MT-ATP6	Mitochondrial	1	0.1%
MT-ND1	Mitochondrial	1	0.1%
MT-ND6	Mitochondrial	1	0.1%
MT-TP	Mitochondrial	1	0.1%
MT-TS2	Mitochondrial	1	0.1%
NPHP1	Autosomal Recessive	1	0.1%
OPN1LW	X-linked Recessive	1	0.1%
OPN1MW	X-linked Recessive	1	0.1%
PDE6A	Autosomal Recessive	1	0.1%
PDE6G	Autosomal Recessive	1	0.1%
PEX10	Autosomal Recessive	1	0.1%
PRCD	Autosomal Recessive	1	0.1%
PRPF3	Autosomal Dominant	1	0.1%
PRPS1	X-linked Recessive	1	0.1%
RBP3	Autosomal Recessive	1	0.1%
RET	Autosomal Dominant	1	0.1%
RLBP1	Autosomal Recessive	1	0.1%
RPGRIP1	Autosomal Recessive	1	0.1%
SAG	Autosomal Dominant	1	0.1%
SCAPER	Autosomal Recessive	1	0.1%
SLC52A2	Autosomal Recessive	1	0.1%
TOPORS	Autosomal Dominant	1	0.1%
TTLL5	Autosomal Recessive	1	0.1%
VHL	Autosomal Dominant	1	0.1%

Gene	Inheritance Pattern	Frequency	% of participants
VPS13B/COH1	Autosomal Recessive	1	0.1%

This table lists all 118 unique genes identified among 673 participants with likely molecular diagnoses, representing a total of 675 gene-level diagnoses. 2 participants had dual molecular diagnoses with *ABCA4* and *PRPH2*. Although 676 participants had positive results (likely molecular diagnoses), 3 were excluded due to unavailable gene-level data. Frequencies represent the number of participants in whom each gene was implicated; participants with dual diagnoses are included in both relevant gene rows.
