# Supplementary material for: Disease and Participant-Related Correlates of Genetic Testing Completion for Hereditary Eye Disorders in a Cohort of over 1400 Patients
Source: Ophthalmol Sci. 2026 May 8;6(7):101218. doi: 10.1016/j.xops.2026.101218 (PMC13292590; doi:10.1016/j.xops.2026.101218)
Supplement: Supplemental Table 8 [file mmc9.pdf]

Supplemental Table 8. Comparison of putative clinical diagnoses between Black and White participants.

**Putative clinical diagnosis**	**White (n)**	**White (%; n=902)**	**Black (n)**	**Black (%; n=350)**
Retinitis Pigmentosa	278	30.82%	130	37.14%
Stargardt disease	155	17.18%	60	17.14%
Pattern Dystrophy	87	9.65%	15	4.29%
Cone Dystrophy	60	6.65%	29	8.29%
Cone-Rod dystrophy	56	6.21%	28	8%
Usher syndrome	49	5.43%	10	2.86%
Unspecified macular dystrophy	24	2.66%	9	2.57%
Best disease (e.g. Bestrophinopathy & Best vitelliform macular dystrophy)	23	2.55%	4	1.14%
L-ORD (late-onset retinal degeneration)	18	2%	3	0.86%
Oculocutaneous Albinism	18	2%	5	1.43%
Hereditary optic neuropathy	15	1.66%	15	4.29%
Retinoschisis	14	1.55%	6	1.71%
Choroideremia	13	1.44%	3	0.86%
Achromatopsia	10	1.11%	6	1.71%
Unspecified retinal dystrophy	10	1.11%	5	1.43%
Stickler syndrome	9	1%	3	0.86%
Mitochondrial retinal dystrophy	8	0.89%	1	0.29%
LCA/SEORD	7	0.78%	3	0.86%
Pseudoxanthoma elasticum	6	0.67%	1	0.29%
Occult macular dystrophy	5	0.55%	3	0.86%
Adult-onset vitelliform macular dystrophy	4	0.44%	0	0%
Bardet-Biedl Syndrome (Laurence-Moon)	4	0.44%	1	0.29%
Blue Cone Monochromacy	4	0.44%	1	0.29%
Congenital Stationary Night Blindness	4	0.44%	1	0.29%
LCHAD-related retinitis pigmentosa	3	0.33%	0	0%
Nyctalopia	3	0.33%	1	0.29%
Enhanced S-Cone Syndrome	2	0.22%	0	0%
Gyrate Atrophy	2	0.22%	0	0%
Hereditary retinal vascular disorder	2	0.22%	1	0.29%
North Carolina macular dystrophy	2	0.22%	1	0.29%
Alport syndrome-related retinal dystrophy	1	0.11%	1	0.29%
Batten disease	1	0.11%	0	0%
Biette Crystalline Corneoretinal Dystrophy	1	0.11%	0	0%
Central areolar choroidal dystrophy (CACD)	1	0.11%	0	0%
Methylmalonic acidemia-related retinal dystrophy	1	0.11%	0	0%
Sorsby fundus dystrophy	1	0.11%	0	0%
Von Hippel-Lindau syndrome	1	0.11%	0	0%
SCA7-related retinal dystrophy (Spinocerebellar ataxia 7)	0	0%	4	1.14%

Frequencies and percentages are column-based within each racial group.
