# Supplementary material for: Disease and Participant-Related Correlates of Genetic Testing Completion for Hereditary Eye Disorders in a Cohort of over 1400 Patients
Source: Ophthalmol Sci. 2026 May 8;6(7):101218. doi: 10.1016/j.xops.2026.101218 (PMC13292590; doi:10.1016/j.xops.2026.101218)
Supplement: Supplemental Table 9 [file mmc10.pdf]

Supplemental Table 9. Comparison of gene frequencies among White (n=468) and Black (n=100) participants with likely molecular diagnoses. Only genes identified in at least one participant are shown. Percentages are calculated within racial groups.

Gene Identified	Frequency (White)	Percent (White; n=468)	Frequency (Black)	Percent (Black; n=100)
ABCA4	112	23.93%	29	29%
USH2A	55	11.75%	3	3%
PRPH2	25	5.34%	4	4%
RHO	24	5.13%	2	2%
BEST1	18	3.85%	1	1%
CHM	12	2.56%	3	3%
RP1	10	2.14%	4	4%
RPGR	10	2.14%	3	3%
PRPF31	8	1.71%		
CRX	7	1.5%	1	1%
MAK	7	1.5%		
NR2E3	7	1.5%		
RS1	7	1.5%	2	2%
BBS1	6	1.28%	1	1%
MT-TL1	6	1.28%		
PCDH15	6	1.28%	1	1%
CDH23	4	0.85%	1	1%
CNGA1	4	0.85%		
CNGB3	4	0.85%	1	1%
CRB1	4	0.85%	4	4%
SNRNP200	4	0.85%		
TULP1	4	0.85%	1	1%
ADGRV1	3	0.64%		
C1QTNF5	3	0.64%		
CERKL	3	0.64%		
EYS	3	0.64%	2	2%
FAM161A	3	0.64%		
FLVCR1	3	0.64%		
HADHA	3	0.64%		
IMPDH1	3	0.64%		
IMPG2	3	0.64%		
NYX	3	0.64%		
OPA1	3	0.64%	1	1%
PDE6B	3	0.64%	3	3%
PRDM13	3	0.64%		
PROM1	3	0.64%	2	2%
RPE65	3	0.64%	2	2%
ABCA4+PRPH2	2	0.43%		
ABCC6	2	0.43%		
CEP290	2	0.43%	1	1%
CLN3	2	0.43%	1	1%
CNGA3	2	0.43%	3	3%
COL2A1	2	0.43%	2	2%
GPR143	2	0.43%		
GUCA1A	2	0.43%		
IFT140	2	0.43%		
KCNV2	2	0.43%		
MT-ND4	2	0.43%	1	1%

Gene Identified	Frequency (White)	Percent (White; n=468)	Frequency (Black)	Percent (Black; n=100)
MYO7A	2	0.43%		
NRL	2	0.43%		
OPN1LW/OPN1MW	2	0.43%	1	1%
RP1L1	2	0.43%	2	2%
RP2	2	0.43%		
TYR	2	0.43%		
USH1C	2	0.43%		
AFG3L2	1	0.21%		
ARMS2	1	0.21%		
BBS2	1	0.21%		
C8ORF37	1	0.21%		
CACNA2D4	1	0.21%		
CEP78	1	0.21%		
CLN1	1	0.21%		
CLRN1	1	0.21%		
COL4A5	1	0.21%		
CTNNA1	1	0.21%		
CYP4V2	1	0.21%		
Col11A1	1	0.21%		
EXOSC5	1	0.21%		
FZD4	1	0.21%		
GNPTG	1	0.21%		
GPR98	1	0.21%		
GUCY2D	1	0.21%	2	2%
HGSNAT	1	0.21%		
KIZ	1	0.21%		
LRP2	1	0.21%		
MFSD8/CLN7	1	0.21%		
MT-ATP6	1	0.21%		
MT-ND6	1	0.21%		
MT-TS2	1	0.21%		
NPHP1	1	0.21%		
OAT	1	0.21%		
OCA2	1	0.21%	2	2%
OPN1LW	1	0.21%		
OPN1MW	1	0.21%		
PDE6A	1	0.21%		
PDE6G	1	0.21%		
PRCD	1	0.21%		
PRPF3	1	0.21%		
PRPF8	1	0.21%	1	1%
PRPS1	1	0.21%		
RBP3	1	0.21%		
RET	1	0.21%		
RPGRIP1	1	0.21%		
SAG	1	0.21%		
SLC52A2	1	0.21%		
TOPORS	1	0.21%		
VHL	1	0.21%		
VPS13B/COH1	1	0.21%		
ACO2			2	2%
BBS4			1	1%
CACNA1F			1	1%

Gene Identified	Frequency (White)	Percent (White; n=468)	Frequency (Black)	Percent (Black; n=100)
IMPG1			1	1%
MT-ND1			1	1%
MT-TP			1	1%
SCA7			6	6%
