# Supplementary material for: Disease and Participant-Related Correlates of Genetic Testing Completion for Hereditary Eye Disorders in a Cohort of over 1400 Patients
Source: Ophthalmol Sci. 2026 May 8;6(7):101218. doi: 10.1016/j.xops.2026.101218 (PMC13292590; doi:10.1016/j.xops.2026.101218)
Supplement: Supplemental Table 10 [file mmc11.pdf]

Supplemental Table 10. Comparison of clinical and genetic testing characteristics between non-Hispanic White and Other race participants with genetic eye disorders. Data presented as median (IQR). Rates of genetic testing completion and likely molecular diagnosis by race are reported separately in **Supplemental Table 3** and **Table 3**, respectively.

Parameter	White (N=902)	Other (N=106)	Raw p-value	Adjusted p-value
Age of symptom onset	30 (IQR 13-49)	19.5 (IQR 6-34.8)	M-W p=6.0e-05	4.2e-04*
Age at presentation	46 (IQR 29-59)	36 (IQR 20.2-45.8)	M-W p=3.4e-06	2.7e-05*
Symptom duration to presentation	5 (IQR 1-20.8)	8 (IQR 2-18)	M-W p=0.358	0.36
Baseline BCVA, better-seeing eye	0.19 (IQR 0-0.544)	0.398 (IQR 0.097-0.856)	M-W p=0.001	0.008*
Baseline BCVA, worse-seeing eye	0.398 (IQR 0.097-0.796)	0.544 (IQR 0.301-1)	M-W p=0.001	0.008*
Follow-up BCVA, better-seeing eye	0.301 (IQR 0.097-0.875)	0.61 (IQR 0.176-1)	M-W p=0.011	0.034*
Follow-up BCVA, worse-seeing eye	0.602 (IQR 0.204-1.176)	0.799 (IQR 0.398-1.277)	M-W p=0.03	0.06
Duration of follow-up	6 (IQR 2-12)	2.5 (IQR 1-7)	M-W p=2.5e-08	2.3e-07*
Time from presentation to GT completion (years)	2 (IQR 0-8)	1 (IQR 0-3)	M-W p=0.003	0.012*

*Adjusted p<0.05 (Holm-Bonferroni correction for multiple comparisons)
Abbreviations: BCVA best-corrected visual acuity (logMAR); GT genetic testing; IQR interquartile range; M-W Mann-Whitney U test
